# First trimester lower abdominal cysts as early predictor of anorectal malformations

**DOI:** 10.1007/s40477-022-00744-6

**Published:** 2022-12-23

**Authors:** Marta Erculiani, Emanuele Trovalusci, Cinzia Zanatta, Maria Sonia De Lorenzis, Elisa Filippi, Gabriella Bracalente, Paola Midrio

**Affiliations:** 1 Pediatric Surgery Unit, Department of Women’s and Children’s Health, University of Padua, Padua, Italy; 2 Prenatal Diagnosis Unit, Obst. & Gyn. Department, Ca’ Foncello Hospital, Treviso, Italy; 3 Pediatric Surgery Unit, Ca’ Foncello Hospital, AULSS 2, Treviso, IT Italy

**Keywords:** Anorectal malformation, Prenatal diagnosis, Ultrasound, Lower abdomen cyst, First trimester

## Abstract

**Introduction:**

Prenatal ultrasound diagnosis of anorectal malformations (ARMs) is challenging and often missed as direct visualization of the anal sphincter is not routinely performed, plus the technique is operator-dependent and inaccurate, also in expert hands. Other indirect signs, such as rectosigmoid overdistension or intraluminal calcifications, are occasionally present in late pregnancy. The detection of a cyst of the lower abdomen in the first trimester may be an early sign of ARM. Here we reported our experience and a review of the literature of such cases.

**Material and methods:**

Isolated cases of lower abdomen cysts encountered in the first trimester at the Prenatal Diagnosis Unit during the last 5 years were retrieved and compared with those found in literature. Post-natal clinical data were analyzed to check the presence and type of malformations.

**Results:**

A total of three cases of lower abdomen cysts were found in our center and 13 in literature. In our case series all the cysts spontaneously regressed and were no longer visible since the second trimester of pregnancy, while in literature this was reported in only 4 out of 13 cases. ARM was confirmed in all patients at birth or post-mortem.

**Conclusions:**

The finding of a lower abdomen cyst during the first trimester of pregnancy could be an early predictive sign of ARM, even if it disappears during pregnancy. In these cases, we suggest mentioning to the parents the possibility of an ARM during the counseling and to refer the couple to a Colorectal Center.

**Graphical Abstract:**

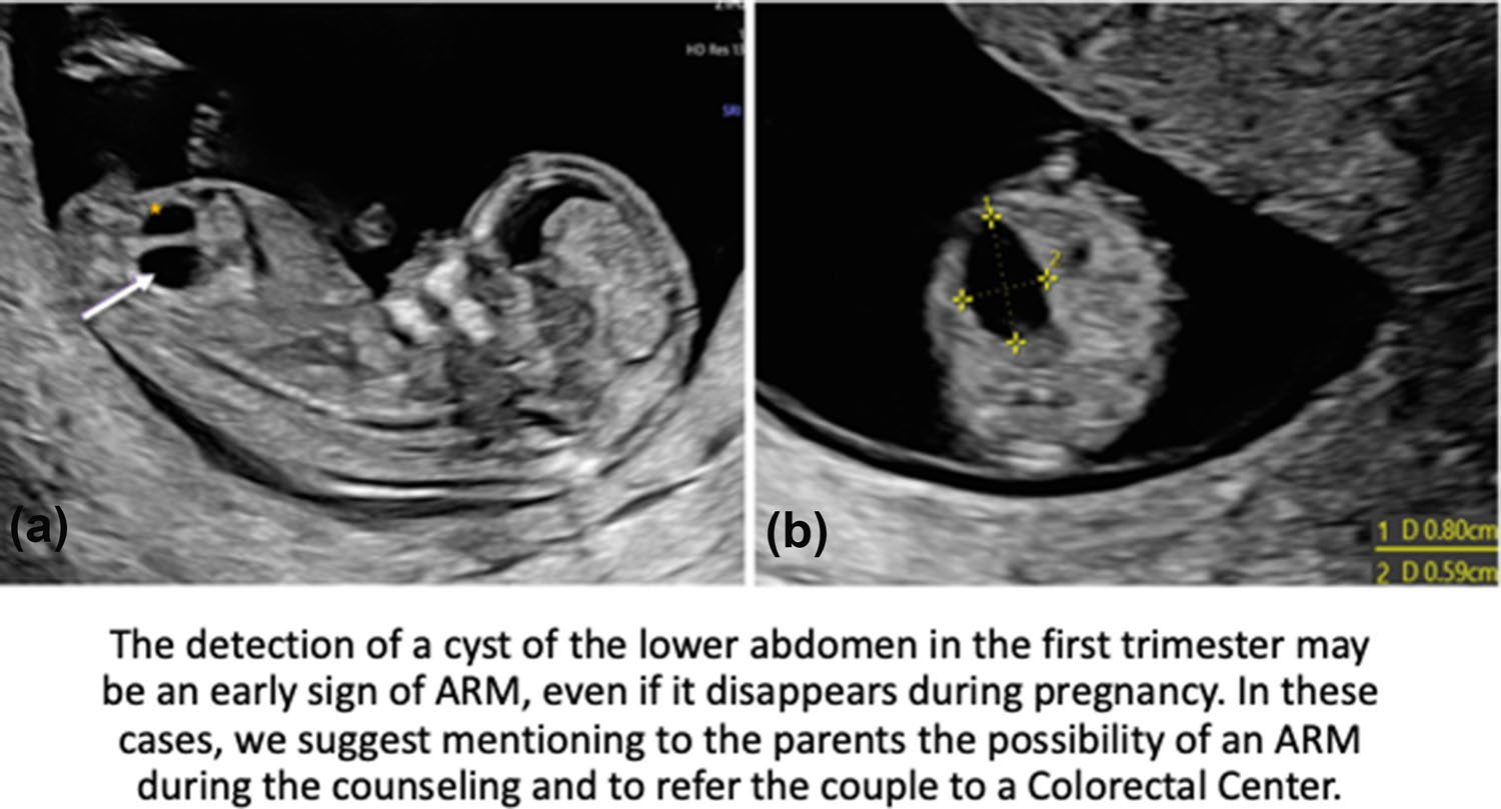

## Introduction

Anorectal malformations (ARM) are a complex spectrum of congenital defects of the distal bowel occurring in 1:2000–5000 live births [1]. Despite the extensive use of prenatal screening sonography (US) for detecting fetal conditions, most ARM are first diagnosed at birth with an incidence of missed diagnosis that may range from 26 to 88% [2]. In normal fetuses, the anal sphincter can be observed at sonography since late second trimester of pregnancy as a target-like image [3]; the absence of this target is the foremost direct imaging sign suggesting ARM. However, the visualization of the anal sphincter and the study of perineal region is not required at the screening US, and this could justify the poor rates of prenatal detection. Moreover, the technique is highly operator-dependent and inaccurate also in expert hands. Occasionally ARM can be suspected by indirect US signs, in particular distal bowel distension and intraluminal calcification [4], but they typically appear in late pregnancy.

Review of the literature has shown only a few cases of isolated ARM suspected in the first trimester, described at ultrasound mainly as cystic or tubular structures of the lower abdomen. Here we reported our experience with three such cases.

## Materials and methods

Isolated cases of lower abdomen cysts (beneath the umbilical cord) encountered in the first trimester at the Prenatal Diagnosis Unit of the Ca’ Foncello Hospital, Treviso – Italy, during the last 5 years were retrieved. Sonographic characteristics of the fetuses and cystic lesions were retrieved, as well as mothers’ clinical data, pregnancy courses, and post-natal outcome of newborns. Ultrasound exams were performed by the same team using the G.E. Voluson E10^®^ Ultrasound Machine, with follow-up every 1–2 weeks after the diagnosis of a lower abdomen cyst till its complete regression.

We used the following combination of keywords to search analogous case reports and series on Pubmed: “first trimester”, “prenatal diagnosis”, “anal atresia” “anorectal malformation”, “anal sphincter”, prenatal ultrasound”, “cystic anomalies “, “pelvic cyst”, and “abdominal cyst”. Cases presenting OEIS complex (omphalocele-exstrophy-imperforate anus-spinal defects) were excluded because the cyst was due to omphalocele.

## Results

A total of three cases of lower abdominal cysts were found in our records. Characteristics are summarized in Table 1 and compared with 13 cases retrieved in literature.

**Table 1:** Summary of ARM cases suspected during the first trimester of pregnancy

Study	N° patients	Fetal Gender	Gestational Age at Diagnosis (weeks)	Ultrasound aspect of the lower abdominal lesion	Genetic Assessment	Term of Pregnancy	Type of ARM (Postpartum/Postmortem diagnosis)
Carroll [11]	1	F	12	Multiple cysts	Normal 46XX	2nd trimester	Female pseudohermaphroditism with agenesis of the urethra, vagina, and rectum
Lam [12]	1	M	12	Sausage-shaped cystic mass (11 × 6 × 6 mm)	Normal 46XY	2nd trimester	Anal atresia, malrotation of the gut, dilated sigmoid colon and rectum and a perimembranous ventricular septal defect
Taipale [13]	1	M	12 + 4	Hypoechogenic cystic mass (14 × 7 × 8 mm)	/	Live born	Anal atresia with fistula
Gilbert [14]	1	M	12 + 4	Cyst with a distal tapered appearance	/	Live born	Imperforate anus horseshoe kidney and low termination of the spinal cord at the third lumbar vertebral body
Chen [15]	1	F	12	Multiple dilated bowel loops	46 XX	Spontaneous miscarriage (18 weeks)	Anorectal atresia, arthrogryposis multiplex, CoA, univentricular heart
Novikova [16]	2	F M	11 + 2 11 + 3	Dilated bowel in the lower abdomen (10 × 2 mm) Hypoechogenic ovoid structure (19 × 10 mm)	Normal 46 XX Trisomy21 XY	18 weeks 12 weeks	Anorectal atresia + multiple anomalies compatible with Fraser syndrome Anorectal malformation in Down syndrome
Santos [17]	1	M	13	Cystic formation	Normal 46 XY	1st trimester	VACTERL syndrome
Bronshtein [18]	2	/ /	13 + 4 14	Distended sigma Distended sigma	/ /	1st trimester Live born	Anal atresia and VACTERL Anal atresia
Correia [19]	1	M	12	Hypoechoic tubular-shaped cyst	Normal 46 XY	23 weeks	Agenesis of the distal portion of the rectum and the anus, rectovesical fistula, glandular hypospadias
Liberty [8]	1	M	13 + 1	Tubular cystic structure (7.5 × 8.5 × 4 mm)	Normal 46 XY	23 weeks	High type ARM
Ples [20]	1	M	11 + 3	Anechoic tubular structure	Arr (1–22) × 2, (XY) × 1	Live born	Perineal fistula
Our experience	3	M F M	12 + 4 13 12 + 6	Cystic structure (8 mm) Cystic structure (5 mm) Cystic structure (8 mm)	Normal 46 XY Normal 46 XX Normal 46 XY (Prenatal detection of MNX1 gene mutation)	Live born Live born (pPROM) Live born	ARM without fistula Rectovestibular fistula Bucket-handle in Currarino Syndrome (altered sacrum, tethered cord)

Abbreviations: *pPROM* preterm premature rupture of membranes

### Case 1

A 31-years-old healthy woman underwent an ultrasound examination at 12 weeks and 4 days of gestation. On transabdominal US an anechoic cystic structure measuring 8 mm and posterior to the bladder was identified in the lower abdomen. From 14 weeks the lesion was no longer detectable. No other fetal malformations were detected during the follow-up. Karyotype analysis was normal (46,XY). The woman had a normal spontaneous vaginal delivery at 38 weeks of gestation and the child weighed 3280*g*. At birth, the definitive diagnosis of ARM with no fistula was made. Screening for associated anomalies was negative and the patient underwent colostomy at birth and anorectal reconstruction (PSARP) 5 months.

### Case 2

A 30-years-old healthy woman underwent an ultrasound examination at 13 weeks of gestation. On transabdominal US an anechoic cystic structure measuring 5 mm with hyperechoic walls was identified in the lower abdomen posterior to the bladder. From 19 weeks the structure was no longer detectable. No other malformations were detected and karyotype analysis was normal (46,XX). Due to preterm premature rupture of membranes (pPROM) the woman had a vaginal delivery at 36 weeks of gestation. The child was a female weighing 2410*g* and the diagnosis of rectovestibular fistula was made at perineal inspection. Screening for associated anomalies was negative and the patient underwent PSARP at 2 days of life.

### Case 3

**Fig. 1:**
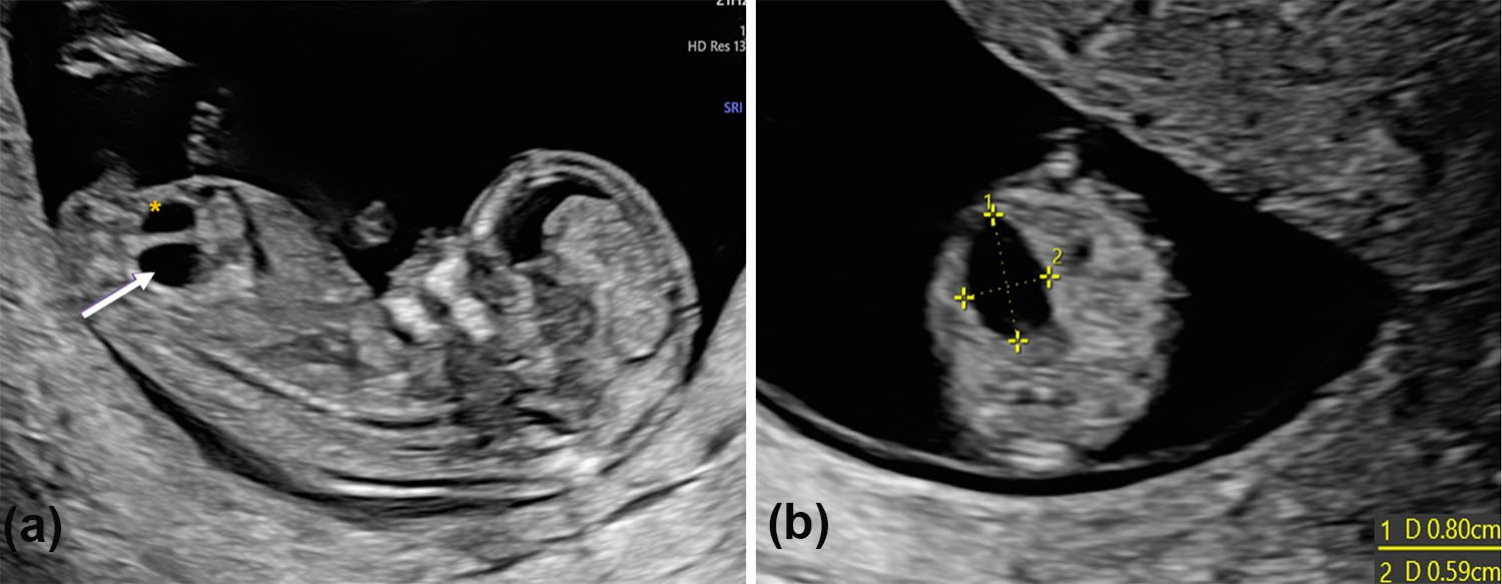
**A** Sagittal view of a lower abdomen cyst (arrow) detected at first trimester US behind the bladder (*); **B** Axial view and measures of the cyst

**Fig. 2:**
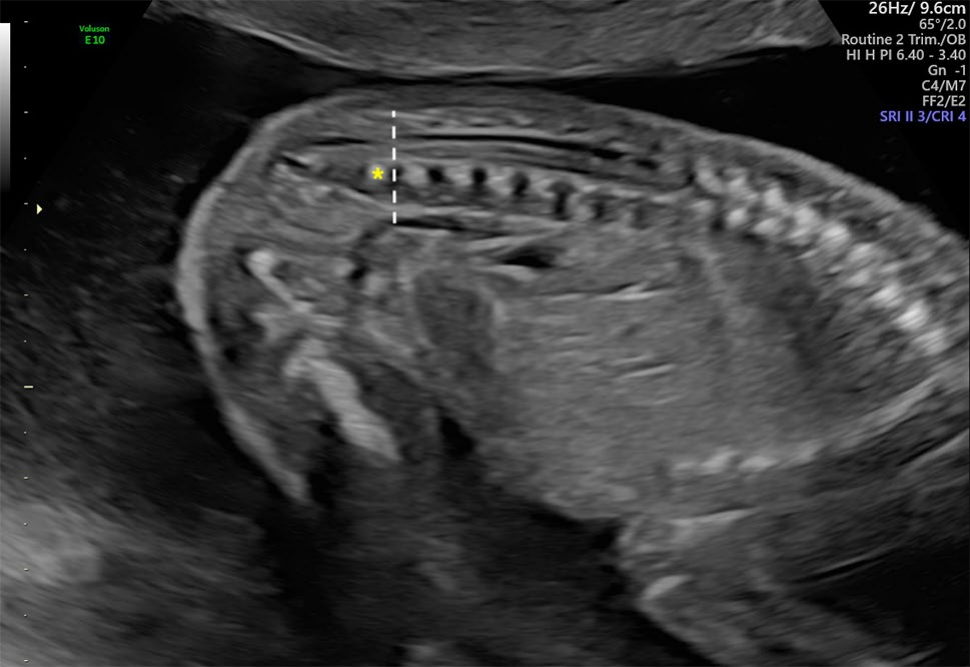
US image of a suspected tethered cord. Dotted line indicates the position of the tip of the conus, which seems to end at the superior L5 margin (*)

A 39-years-old patient with medical history of anterior sacral meningocele, anal stenosis and a scimitar sacrum performed the first ultrasound examination at 12 weeks and 6 days of gestation. On transabdominal US a cystic structure measuring 8 mm (Fig. 1) was identified in the right lower abdomen. From 16 weeks the structure was no longer detectable. At 20 weeks an altered sacrum and possible tethered cord were detected (Fig. 2). Due to maternal history, a genetic analysis in the mother and amniocentesis were performed to search for MNX1 gene mutation; diagnosis of Currarino syndrome was made in both the mother and fetus. Karyotype analysis of the fetus was 46, XY. Due to sacrum and spinal anomalies of the mother, a cesarean section at 38 weeks and 5 days of gestation was performed. The child was a male weighing 3170*g* and a diagnosis of perineal fistula with a bucket handle was performed. The newborn underwent PSARP at 2 days of life.

## Discussion

Prenatal diagnosis of ARM is challenging, especially during the first trimester of pregnancy. Most ARMs continue to be diagnosed at birth; in literature, it is reported that 16% of cases are detected in utero—considering the whole pregnancy period—and more frequently these are complex types of ARM, especially cloaca and exstrophy of cloaca [4].

Anal sphincter development begins at the end of the first trimester; Bourdelat et al. [5] were the first to describe the sonographic features and measurements of the normal anal sphincter in fetuses. The three muscular components of the anal sphincter are usually present since the 14–15th weeks and continues to develop throughout the second semester, reaching the full maturation between 28 and 30 weeks. From the 22–23 weeks onwards, the anal sphincter can be visualized on sonography as an oval hypoechogenic structure surrounding the central echogenic anal mucosa [3]. These findings were subsequently confirmed by Moon et al. [6], who reported a visualization rate of > 90% between 23 and 34 weeks of gestation, but significantly lower (0–60%) between 16 and 22 weeks, confirming that the missed visualization of the anal sphincter at first trimester US cannot be considered diagnostic. Moreover, the direct observation of the anal sphincter in not always performed during prenatal US.

Indirect signs suspicious for ARM, such as the presence of intraluminal calcification and rectosigmoid distension, are more frequently described in literature. Intraluminal calcifications may form when the calcium-phosphate present in the urine causes calcification of the meconium, meaning a communication between the urinary and intestinal tracts is present. This could be explained by the effect of some digestive enzymes that accumulate within the abnormal or absent fetal anal canal [7]. Anyway, these indirect signs of ARM are a rare event, frequently observed at the end of the second trimester and during the third one.

According to our systematic review of the literature, and similarly to our experience, an ARM was diagnosed during the first trimester of pregnancy in only 13 cases thanks to the finding of anechoic cystic structures. These are the US representations of dilated bowel loops filled with liquid, maybe due to the progressive accumulation of intestinal content in the rectum that ends blindly [8]

When a cystic mass in the fetal abdomen is observed, it prompts differential diagnosis with conditions other than ARM. The most common causes for fetal intra-abdominal cysts are ovarian cysts, gastrointestinal cystic duplication, hepatic and biliary cysts, meconium pseudocysts, mesenteric cysts, adrenal cysts, splenic cysts, bowel dilatation secondary to obstruction, hydrometrocolpos, and other conditions affecting the urinary system. Most of these, such as ovarian cysts and gastrointestinal cysts, are predominantly found during the third trimester of pregnancy. Magnetic Resonance Imaging (MRI) could be helpful to better characterize the nature of fetal abdominal cysts [9], but in our patients this was not performed as the lesions spontaneously regressed during the second trimester.

Similarly to our cases, in 4 out of 13 fetuses the lesions spontaneously disappeared later in pregnancy. This phenomenon could be explained by the decompression of the distended colon through a fistula [10]. On the contrary, in two cases the cystic structure resulted in a dilated stomach and a megacyst, respectively. In the other fetuses the cyst was visible through the entire pregnancy.

Bischoff et al. [4] stated that prenatal diagnosis of cloaca and other complex ARM could be more frequently suspected when US images show the presence of cystic abdominal masses associated with urological abnormalities. We observed that the prenatal finding of a lower abdomen cystic lesion in the first trimester could be a predictable sign of any type of ARM, independently from its anatomical complexity.

In our series, one case was part of Currarino syndrome, confirmed postnatally by genetic analysis. This emphasizes the importance of an early US sign to predict ARM and proceed with further investigations to identify other possible fetal anomalies, refer the mother to a tertiary care hospital, and provide specialist pregnancy and genetic counseling, also in terms of termination or continuation of pregnancy.

## Conclusions

In conclusion, direct signs of ARM are not detectable in the first trimester of pregnancy due to the late development and difficult evaluation of the perianal muscular complex. However, ARM should be considered in differential diagnosis when a lower abdomen cyst is found during the first trimester, even if it disappears during pregnancy. Unfortunately, the absence of prospective studies and the few cases described in literature make this premature finding an inadequate diagnostic sign; however, this justify a careful US prenatal evaluation of the anal dimple from the late second and third trimester and, in case of anomaly, proper counselling should be offered to the mother, considering the morbidity, pathologic associations and impaired outcome of ARM.

## Data Availability

All data generated or analyzed during this study are included in this published article.
